# Healthy aging in place during the pandemic in Northern Italy

**DOI:** 10.1093/eurpub/ckac131.074

**Published:** 2022-10-25

**Authors:** N Paone, S Mairhofer

**Affiliations:** 1 Applied Social Sciences, Hochschule München University of Applied Sciences, Munich, Germany; 2 Faculty of Education, Free University of Bozen - Bolzano, Bolzano, Italy

## Abstract

Many elderly people would like to stay in their own homes as long as possible. Therefore, a focus on enabling factors for a healthy aging in place in needed. Italy was the first European country to be hit hard by the pandemic. These had an impact on people’s everyday lives, on social participation and freedom of movement for all sections of the population. But especially elderly people were considered a risk group and were urged to leave their homes as little as possible. The project aimed to analyse the situation of elderly people in South Tyrol (Northern Italy), focusing on the characteristics of enabling factors for a healthy ageing in place. The main research question was: What kind of enabling factors ensure a healthy aging in place during the pandemic? Using a mixed-methods-approach, we conducted 10 semi-structured interviews (experts: social workers, health professionals, responsible persons from senior associations, ...) analysed by qualitative content analysis and a quantitative questionnaire (536 respondents, aged 60 to 101 still living in their own home) from 2020 to 2021. The questionnaire was distributed in digital and analogue form to reach a wider study group and to facilitate access to the research group despite the infection control measures or technical challenges. The results show that there were numerous changes in the everyday life of elderly people during the pandemic, which were described as particularly important for a healthy ageing in place. Based on the answers to the pandemic-related restrictions, 6 categories could be identified: Loneliness versus desire for social contact, mobility, emotions, needs, opportunities, restrictions. To be able to guarantee healthy ageing in place, we need to examine and promote these enabling factors in the long term.

**Key messages:**

• A focus on enabling factors for a healthy aging in place in needed.

• There were numerous changes in the everyday life of elderly people during the pandemic, which were described as particularly important for a healthy ageing in place.

